# Study of Deformation Stability during Semi-Dieless Drawing of Ti-6Al-4V Alloy Wire

**DOI:** 10.3390/ma12081320

**Published:** 2019-04-23

**Authors:** Kaisong Li, Zhangke Wang, Xuefeng Liu

**Affiliations:** 1 Department of materials processing engineering, School of Materials Science and Engineering, University of Science and Technology Beijing, Beijing 100083, China; ksonglee@xs.ustb.edu.cn (K.L.); wzk326@163.com (Z.W.); 2 Beijing Laboratory of Metallic Materials and Processing for Modern Transportation, University of Science and Technology Beijing, Beijing 100083, China

**Keywords:** Ti-6Al-4V alloy wire, semi-dieless drawing, stability

## Abstract

A semi-dieless drawing technology has the advantages of producing a large deformation in a single pass and achieving high-precision dimensions of the finished products. However, instabilities can easily occur in a technique with a large amount of deformation, resulting in its failure. Herein, the deformation behavior of a wire during semi-dieless drawing is studied by finite element simulations. The instability mechanism of the semi-dieless drawing is proposed and validated by experiments. The experiments are conducted under the following conditions: a heating temperature of 950 °C; a distance between the die and heating coil of 20 mm; a feeding speed of 0.25 mm/s; a drawing speed range of 0.38–0.53 mm/s, and a die diameter range of 1.8–2.4 mm. The results show that by increasing the drawing speed or decreasing die diameter, the diameter fluctuation of the dieless drawn wire increases, and the semi-dieless drawing process easily becomes unstable. The diameter of the entering wire shows a fluctuating increasing trend owing to the variation in the drawing speed, which results in the instability during the semi-dieless drawing. The validity of the finite element model is verified by comparing the numerically predicted value and experimentally measured value of the drawn wire diameter.

## 1. Introduction

Ti-6Al-4V titanium alloy has good corrosion resistance, high-temperature mechanical properties, and biocompatibility; it has been widely used in aerospace, petrochemical, biomedicine, and other fields [1]. Ti-6Al-4V titanium alloy has poor deformation ability at room temperature because of its hexagonal close-packed structure [2]. Traditional die drawing is the main method to prepare Ti-6Al-4V titanium alloy wires [3]. However, this method has the disadvantages of a large drawing force, severe drawing die wear, high consumption of lubricant, and so on [4,5].

Dieless drawing is a flexible forming technology that does not use the conventional drawing die to achieve drawing deformation of a metal workpiece [6]. In the deformation process, an axial tensile force is imposed on a metal workpiece, which is locally heated to an elevated temperature to initiate plastic deformation in a high-temperature zone. Subsequently, the deformed workpiece is cooled to prevent further deformation to achieve products with constant or different cross-sections [7]. Tiernan et al. [8] concluded that dieless drawing has many advantages such as large area reduction in a single pass, small drawing force, and no requirement of a lubricant and drawing die.

Compared with traditional die drawing, the deformation zone in dieless drawing is in an unrestrained state, and its deformation stability is poor, which leads to size fluctuations and even breaking of the finished products [9]. Many researchers have investigated deformation stability in dieless drawing.

Wright et al. [10] believed that the deformation state of a workpiece in dieless drawing was similar to that in a uniaxial tensile test, and the deformation instability in dieless drawing could be explained by the necking theory of uniaxial tension. In a uniaxial tensile process, when the load reaches the maximum value, i.e., *dF* = 0, plastic instability occurs, and a metal material experiences necking [11]. This is the well-known Considerè instability criterion, which is expressed as

(1) $$\frac{d\sigma }{d\varepsilon }=\sigma ,$$

where *σ* is the true stress and *ε* is the true strain.

The constitutive equation of the material is $\sigma =\sigma (\varepsilon ,\dot{\varepsilon },T)$, so that the instability criterion in dieless drawing as derived from the necking theory can be expressed as

(2) $$\frac{d\sigma }{d\varepsilon }=(\frac{\partial \sigma }{\partial \varepsilon })+(\frac{\partial \sigma }{\partial \dot{\varepsilon }})(\frac{\partial \dot{\varepsilon }}{\partial \varepsilon })+(\frac{\partial \sigma }{\partial T})(\frac{\partial T}{\partial \varepsilon }),$$

where σ is the true stress, ε is the true strain, $\dot{\varepsilon }$ is the true strain rate, and *T* is the heating temperature.

Fortunier et al. [12] considered the constant drawing force acting on a wire as the stability criterion, i.e., $\frac{dF}{dt}=0$, and established the condition of stable dieless drawing as

(3) $$\frac{d\dot{\varepsilon }}{dt}=\frac{\dot{\varepsilon }}{\frac{\partial \sigma }{\partial \dot{\varepsilon }}}\left(\sigma -\frac{\partial \sigma }{\partial \varepsilon }\right)-\frac{\frac{\partial \sigma }{\partial T}}{\frac{\partial \sigma }{\partial \dot{\varepsilon }}}\frac{dT}{dt},$$

where $\frac{\partial \sigma }{\partial \varepsilon }$, $\frac{\partial \sigma }{\dot{\varepsilon }}$, and $\frac{\partial \sigma }{\partial T}$ represent the parameters related to strain hardening, strain rate sensitivity index, and temperature coefficients, respectively, and $\sigma =\frac{F}{A}$ and $\frac{dT}{dt}$ represent the stress and temperature distribution, respectively.

Li [13] proposed that when the drawing force remains constant along the axial direction, i.e., $\frac{dF}{dx}=0$, and the transversal area of the deformation zone does not increase along the axial direction, i.e., $\frac{dA}{dx}\leq 0$, the dieless drawing deformation is stable. Thus, the conditions for stable dieless drawing are obtained as

(4) $$\gamma +m+\beta \frac{d\ln T}{d\ln A}\geq 1,$$

where *γ* is the strain hardening coefficient, *m* is the strain rate sensitivity index, *β* is the temperature coefficient, and *A* is the cross-sectional area of the deformation zone.

From Equations (2)–(4), it can be seen that the deformation stability in dieless drawing is closely related to the parameters of the material itself (strain rate sensitivity index *m*, work hardening coefficient *γ*, or work hardening index *n*) and process parameters (heating temperature *T* and strain rate $\dot{\varepsilon }$). Furushima et al. [14] showed that a large strain rate sensitivity index *m* implies good stability in dieless drawing. Milenin [15] showed that a large strain hardening index *n* is beneficial to stable deformation of dieless drawing. When the heating temperature is low, the wire deformation resistance is high. When the heating temperature is high, a wire will undergo dynamic recrystallization and the grains will grow rapidly, leading to poor plasticity [16]. The temperature gradient in the deformation zone also has a significant impact on the deformation stability in dieless drawing. Furushima and Manabe [17] reported that a large temperature gradient corresponds to good stability in dieless drawing. Hwang and Kuo [18] reported that a high drawing speed leads to low deformation stability in dieless drawing.

The above studies reveal the reason for the size fluctuation in dieless drawing, thereby contributing toward improving the size accuracy of the finished products; nevertheless, it is still difficult to fundamentally eliminate size fluctuation. Kawaguchi et al. [19] performed dieless drawing followed by die drawing, which eliminated the size fluctuation of the metal wire being drawn. However, this drawing technology failed to completely utilize the residual heat in dieless drawing; consequently, the efficiency of energy usage was low. In addition, this method is complex. Pre-processing treatments such as pointing and reassembling are required, which complicate the process, and thus, increase the production cost.

Liu et al. [20] proposed a semi-dieless drawing technology, which combined the cooler in dieless drawing with a drawing die and made full use of residual heat in dieless drawing. This new drawing technology achieved a large area reduction by performing dieless drawing and warm die drawing simultaneously, which could improve the diameter accuracy and surface quality of the finished products. Li et al. [21] prepared titanium alloy wires with 33% area reduction by semi-dieless drawing. However, there is a lack of in-depth research on semi-dieless drawing, and the process is prone to instability during large deformations, leading to its failure.

Dieless drawing and die drawing have a mutual influence during semi-dieless drawing, so the instability mechanism of semi-dielss drawing is different form single dieless drawing. In this study, a Ti-6Al-4V titanium alloy wire was used as the raw material to study the deformation behaviour in semi-dieless drawing by finite element (FE) simulations, following which the instability mechanism of the semi-dieless drawing was proposed.

## 2. Finite Element Simulations

To investigate the semi-dieless drawing process, a rigid–plastic FE analysis was performed using DEFORM-3D. For Ti-6Al-4V titanium alloy, the flow stresses at different temperatures in the database of DEFORM-3D were adopted. Semi-dieless drawing is a kind of hot forming technology, so Ti-6Al-4V titanium alloy was assumed to be a rigid–plastic material. The workpiece was 200 mm in length and 3 mm in diameter. The Ti-6Al-4V alloy wire model was meshed by tetrahedral elements and divided into 200,000 elements. The drawing die material was YG6 cemented carbide, which is a rigid material. The diameter of the die was 2 mm and the semi-die angle α was 12°. There was heat conduction between the wire and die; therefore, the die needed to be meshed. The drawing die model was meshed by tetrahedral elements and divided into 20,000 elements. The friction coefficient between the die and wire was 0.2. The initial temperature of the wire, die, and environment was 20 °C. The heat capacity and thermal conductivity of the workpiece are 611 J·kg^−1^·K^−1^ and 6.8 W·m^−1^·K^−1^, respectively. The heat transfer coefficient between the workpiece and drawing die and between the workpiece and air was 11 and 20 W·m^−2^·K^−1^, respectively.

**Figure 1:**
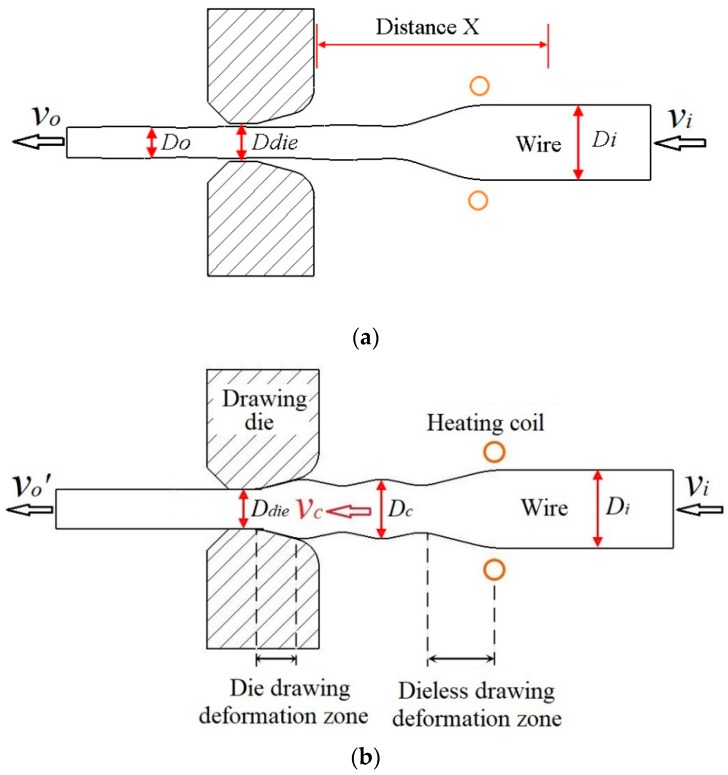
Finite element model of the semi-dieless drawing: (**a**) initial stage, (**b**) semi-dieless drawing stage; *v_i_*—feeding speed, *v_o_*—drawing speed of the dieless drawing, *D_i_*—diameter of the raw wire, *D_o_*—dieless drawn wire diameter, *v_o_*′—drawing speed of the semi-dieless drawing (simply drawing speed for short), *D_die_*—hole diameter of the die, *D_c_*—diameter of the entry wire, *v_c_*—drawing speed of the dieless drawing section, which is also the entering speed of the die drawing section.

The semi-dieless drawing process was divided into two stages, which is shown in Figure 1. The first stage was dieless drawing, which was called the initial stage. According to the volume constant law,

(5) $$v_{i}D_{i}^{2}=v_{o}D_{o}^{2},$$

by setting drawing speed *v_o_* and feeding speed *v_i_*, diameter of the deformed wire *D_o_* is made smaller than diameter of the die *D_die_*. During the second stage, the drawing die was inserted. According to the volume constant law,

(6) $$v_{i}D_{i}^{2}={v_{o}}^{\prime }D_{c}^{2},$$

when reducing drawing speed to *v_o_*′, dieless drawn wire diameter *D_c_* becomes larger than die diameter *D_die_*, and the wire deformation changes from dieless drawing to semi-dieless drawing. For instance, the initial wire diameter was 3 mm, the die diameter was 2.4 mm, the feeding speed was 0.25 mm/s, the drawing speed was 0.42 mm/s, and according to Equation (5), the dieless drawn wire diameter *D_o_* was 2.32 mm, which is smaller than the die diameter. Then the drawing speed was reduced to 0.38 mm/s, and according to Equation (6), the dieless drawn wire diameter *D_c_* was 2.43 mm, which is larger than the die diameter.

Semi-dieless drawing involves dieless drawing and die drawing. During the drawing, the wire diameter changes from *D_i_* to *D_c_* by dieless drawing, and then to *D_die_* by die drawing. The deformation amounts in dieless drawing and die drawing are:

(7) $$\varphi_{dieless}=\frac{D_{i}^{2}-D_{c}^{2}}{D_{i}^{2}},$$

(8) $$\varphi_{die}=\frac{D_{c}^{2}-D_{die}^{2}}{D_{c}^{2}},$$

In this study, we examined the influence of the drawing speed *v_o_*′ and die diameter *D_die_* on the deformation behaviour in a semi-dieless drawing for a constant feeding speed *v_i_*. If the diameter of the die varies, the deformation amount in die drawing will vary when the dieless drawn wire enters the die. Substituting Equation (6) into Equation (8), we get

(9) $$\varphi_{die}=1-\frac{{v_{o}}^{\prime }D_{die}^{2}}{v_{i}D_{i}^{2}},$$

Using Equation (9), the diameter of the die corresponding to different deformation amounts in die drawing can be calculated, as given in Table 1.

The FE simulations were performed using a heating temperature of 950 °C, distance between the drawing die and heating coil of 20 mm, feeding speed of 0.25 mm/s, drawing speed of 0.38–0.53 mm/s, and die diameter of 1.8–2.4 mm.

## 3. Results and Discussion

### 3.1. Stability of Semi-Dieless Drawing

Owing to the unconstrained state of the dieless drawing deformation zone, the diameter of the dieless drawn wire fluctuates. The fluctuation can then be removed by the subsequent die drawing, as shown in Figure 1b. The drawing speed of the dieless drawing section v_c_ is also the entering speed of the die drawing section. Dieless drawing and die drawing have a mutual influence during semi-dieless drawing.

**Figure 2:**
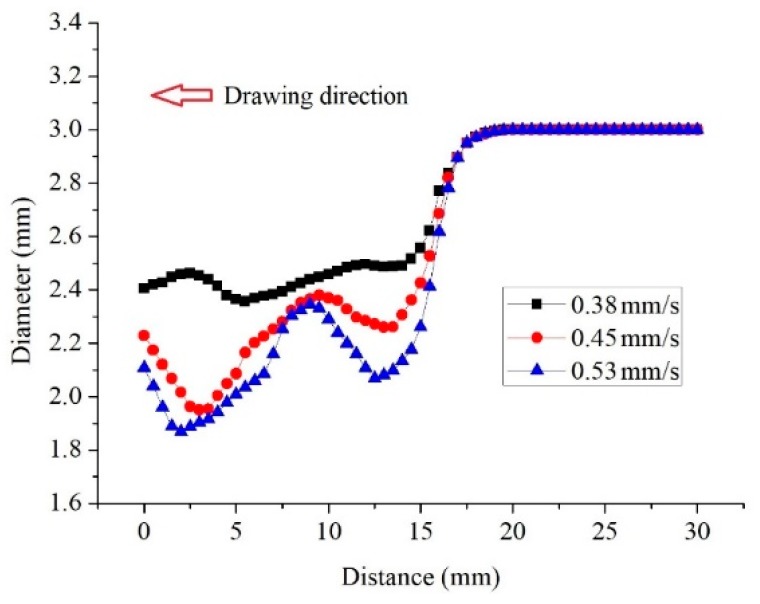
Wire diameter at drawing time *t* = 105 s.

In the initial stage, the wire only undergoes dieless drawing, and the diameter of the dieless drawn wire is smaller than the diameter of the die. There is no contact between the die and dieless drawn wire, so that the die has no influence on the wire diameter. The wire diameter was measured from the entrance of the drawing die to the raw wire, as shown in Figure 1a. The wire diameter at drawing time *t* = 105 s is shown in Figure 2. As can be seen from the figure, with increasing drawing speed, the wire diameter decreases, whereas the diameter fluctuation increases. This is owing to the fact that the diameter of the dieless drawn wire decreases with increasing speed ratio. For a large speed ratio, the stability in dieless drawing is poor and the diameter fluctuation is large. Tiernan and Hillery [22] studied the variation law of a wire diameter during dieless drawing deformation and obtained the same conclusion.

**Figure 3:**
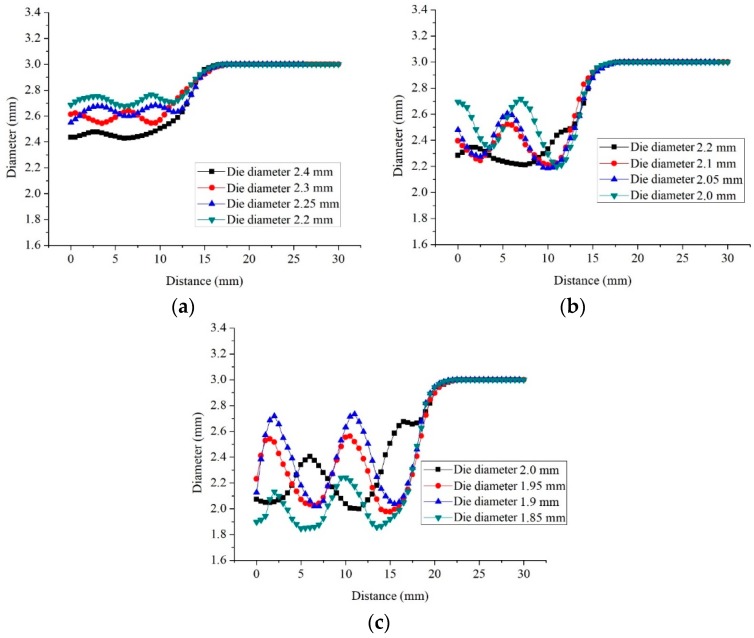
Wire diameter variation at drawing time *t* = 145 s under the condition of feeding speed 0.25 mm/s and drawing speed: (**a**) 0.38 mm/s, (**b**) 0.45 mm/s, (**c**) 0.53 mm/s.

During semi-dieless drawing deformation, the dieless drawn wire enters the die and undergoes die drawing. At this time, the die influences the diameter of the dieless drawn wire. The wire diameter was measured from the entrance of the drawing die to the raw wire, as shown in Figure 1a. The wire diameter variation at drawing time *t* = 145 s is shown in Figure 3. Figure 3a shows the wire diameter for a feeding speed of 0.25 mm/s, drawing speed of 0.38 mm/s, and die diameter of 2.4, 2.3, 2.25, and 2.2 mm, respectively. When the diameter of the die was 2.4 mm, the peak and valley of the wire diameter were 2.44 mm and 2.41 mm, respectively. The wire diameter fluctuation was 0.03 mm, which is small. When the diameter of the die was 2.3 mm, the peak and valley of the wire diameter were 2.63 mm and 2.56 mm, respectively, and the wire diameter fluctuation was 0.07 mm. When the diameter of the die was 2.25 mm, the peak and valley of the wire diameter were 2.68 mm and 2.59 mm, respectively, and the wire diameter fluctuation was 0.09 mm. When the diameter of the die was 2.2 mm, the peak and valley of the wire diameter were 2.77 mm and 2.67 mm, respectively. The wire diameter fluctuation was 0.1 mm, which is large. Therefore, with decreasing die diameter, i.e., increasing the die drawing deformation amount, the wire diameter and its fluctuation increase.

Figure 3b shows the wire diameter for a feeding speed of 0.25 mm/s, drawing speed of 0.45 mm/s, and die diameter of 2.2, 2.1, 2.05, and 2.0 mm, respectively. When the diameter of the die decreased from 2.2 to 2.0 mm, the diameter of the wire increased, and its fluctuation increased from 0.13 to 0.51 mm.

Figure 3c shows the wire diameter for a feeding speed of 0.25 mm/s, drawing speed of 0.53 mm/s, and die diameter of 2.0, 1.95, 1.9, and 1.8 mm, respectively. When the diameter of the die decreased from 2.0 to 1.9 mm, the diameter of the wire increased and its fluctuation increased from 0.4 to 0.71 mm. When the diameter of the die was 1.85 mm, the wire breaks at the die exited as soon as the dieless drawn wire entered the drawing die. Subsequently, the dieless drawing stopped, and the wire diameter fluctuation was 0.39 mm.

**Figure 4:**
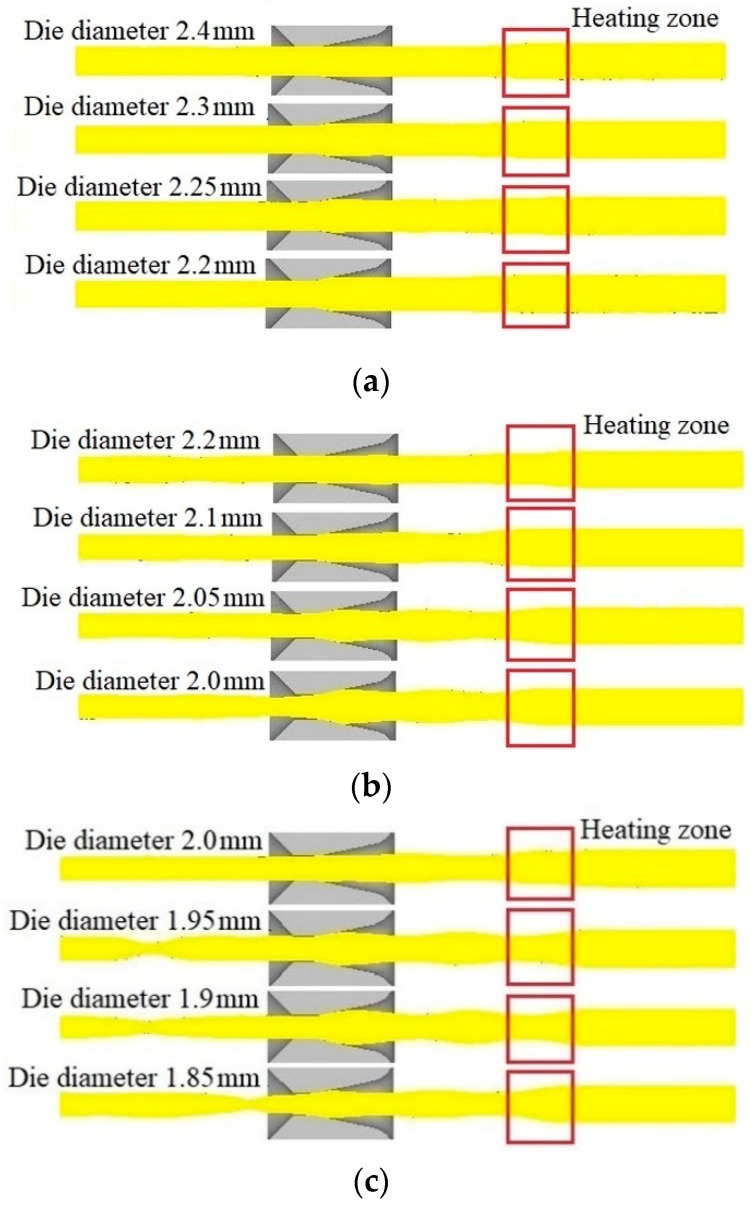
Profile of the wire at drawing time *t* = 145 s under the condition of feeding speed 0.25 mm/s and drawing speed: (**a**) 0.38 mm/s, (**b**) 0.45 mm/s, (**c**) 0.53 mm/s.

Figure 4 shows the profile of the wire at drawing time *t* = 145 s. It can be seen from the figure that when the drawing speed was 0.38 mm/s, the wire size fluctuation was small and the semi-dieless drawing occurred smoothly. When the drawing speed increased to 0.45 and 0.53 mm/s, the wire diameter fluctuation increased, and the wire underwent necking at the die exit. It can also be seen from the figure that when the diameter of the die was large, i.e., the deformation amount in the die drawing was small, the wire diameter fluctuation was small and the semi-dieless drawing occurred smoothly. When the diameter of the die was small, i.e., the deformation amount of the die drawing was large, the wire diameter fluctuated significantly, and the wire appeared to undergo necking at the die exit.

Therefore, during semi-dieless drawing, when the diameter of the die is large, i.e., the deformation amount in the die drawing process is small, the wire diameter and its fluctuation are small, and the semi-dieless drawing process is stable. With decreasing die diameter, i.e., increasing die drawing deformation amount, the wire diameter and its fluctuation increase. Consequently, the stability in the semi-dieless drawing process deteriorates, and the wire is prone to necking or even breaking at the die exit, leading to the failure of semi-dieless drawing. Similarly, when the drawing speed increases, the wire diameter fluctuation also increases, and the deformation stability in the semi-dieless drawing process becomes worse.

### 3.2. Instability Mechanism of Semi-Dieless Drawing

#### 3.2.1. Instability Evolution in Semi-Dieless Drawing

**Figure 5:**
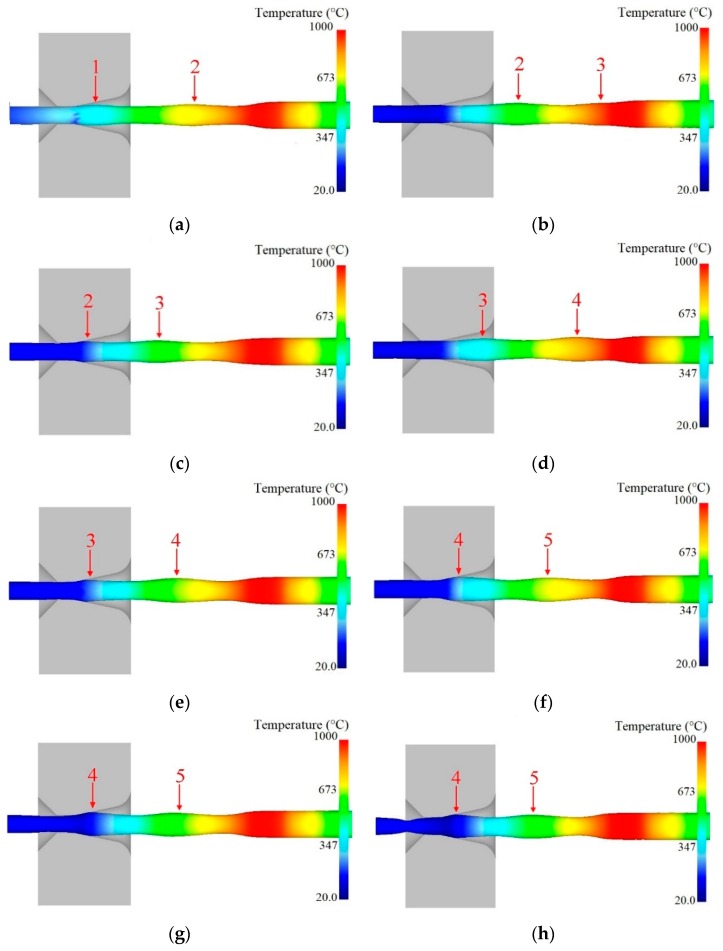
Instability evolution in the semi-dieless drawing process: (**a**) *t* = 135 s, (**b**) *t* = 145 s, (**c**) *t* = 165 s, (**d**) *t* = 175 s, (**e**) *t* = 185 s, (**f**) *t* = 205 s, (**g**) *t* = 210 s, (**h**) *t* = 215 s; 1–5 represent the wave peaks of the wire.

Figure 5 shows the instability evolution during semi-dieless drawing with a feeding speed of 0.25 mm/s, drawing speed of 0.53 mm/s, diameter of the die 2.0 mm, and heating temperature of 950 °C. As can be seen from the figure, when the simulation time was 135 s, there were two fluctuation segments between the dieless and die drawing deformation regions, and the wire diameter at the wave peak was nearly the same, as shown in Figure 5a. When the simulation time was 145 s, the first fluctuation section eliminates the size fluctuation through the die drawing, the second fluctuation section continues to move forward, and the third fluctuation section occurs during dieless drawing, as shown in Figure 5b. When the simulation time was 165 s, the second fluctuation section deformed in the drawing die, the third fluctuation section continued to move forward, and the wire diameter at the third wave peak was larger than that at the second wave peak, as shown in Figure 5c. As deformation progresses, new fluctuation segments will be generated continuously during die drawing of the previous fluctuation segments, and the wire diameter at the wave peak will continue to increase, as shown in Figure 5d–f. When the simulation time was 210 s, the fourth fluctuation section continues to deform in the drawing die. At this point, the drawing stress of the wire at the die exit is larger than the yield strength of the wire, and the wire experiences necking shrinkage at the die exit, as shown in Figure 5g. When the simulation time was 215 s, the necking of the wire continued to increase, the fourth fluctuation section no longer deformed in the drawing die, and semi-dieless drawing stopped, as shown in Figure 5h.

**Figure 6:**
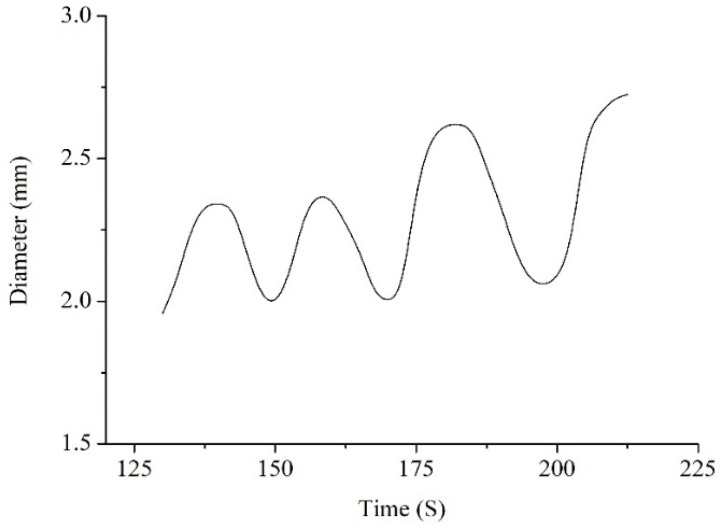
Wire diameter evolution at entrance of the drawing die.

The values of wire diameter at entrance of the drawing die are shown in Figure 6. We can see from the figure that the wire diameter shows a fluctuating increasing trend.

It can be seen that the diameter fluctuation of the dieless drawn wire is small, and it is eliminated by the subsequent die drawing at the beginning of the semi-dieless drawing process. As this process proceeds, the wire diameter shows a fluctuating increasing trend. When the diameter of the wire peak increases to a certain extent, i.e., it exceeds the limit range of the die drawing, the wire experiences necking or even breaking at the die exit, leading to the failure of the semi-dieless drawing process. He et al. [23] reported that the wire diameter fluctuation is basically constant and there is no diameter fluctuating increase when the process parameters remain constant in single dieless drawing. The wire diameter fluctuation can be eliminated by the subsequent die drawing to prepare finished products with high precision [24]. Owing to the interaction between the dieless and die drawing during the process of semi-dieless drawing, the deformation behaviour is different from first dieless drawing and then die drawing.

The above results indicate that instability in semi-dieless drawing is owing to the fact that the diameter of the dieless drawn wire shows a fluctuating increasing trend, which leads to an increase in the die drawing deformation amount. When the die drawing deformation amount exceeds the safe drawing limit, the wire will undergo necking at the die exit, and semi-dieless drawing will fail.

#### 3.2.2. Instability Mechanism of Semi-Dieless Drawing

**Figure 7:**
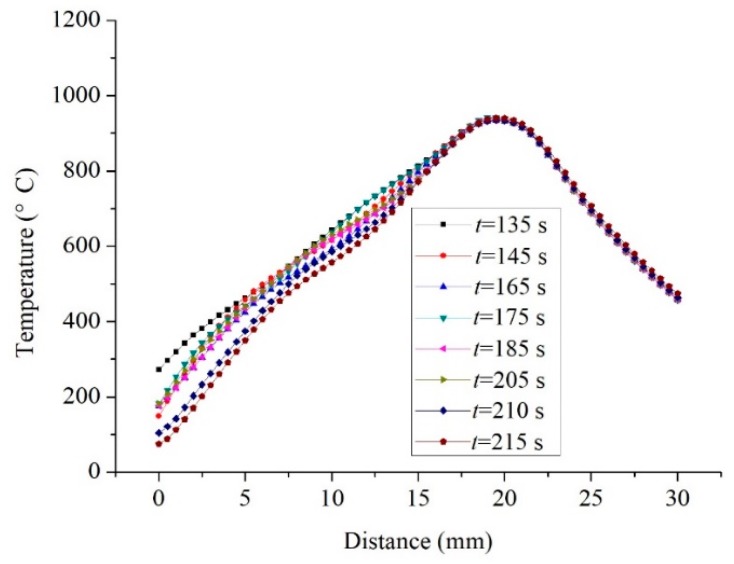
Temperature distribution along the wire axial direction.

Because there is no die constraint in the dieless drawing deformation zone, the wire is prone to size fluctuations and is in an unstable state when the deformation amount is large [25]. Once the raw wire is selected, the heating temperature and drawing speed are the two key factors that influence the stability in the dieless drawing process. The wire temperature was measured from the entrance of the drawing die to the raw wire, as shown in Figure 1a. Figure 7 shows the temperature distribution along the wire axial direction during the semi-dieless drawing (corresponding to Figure 5). It can be seen from the figure that the maximum temperature in the dieless drawing deformation zone is 950 °C, and the temperature gradient is large, which is beneficial for achieving stable dieless drawing. Li et al. [26] studied the influence of the temperature distribution on the stability in dieless drawing and reported the same conclusion. As the semi-dieless drawing process progresses, the temperature distribution in the dieless drawing deformation zone remains stable. Therefore, in the above deformation process, the temperature gradient in the dieless drawing deformation zone is high and stable, which is conducive for stable deformation in semi-dieless drawing.

**Figure 8:**
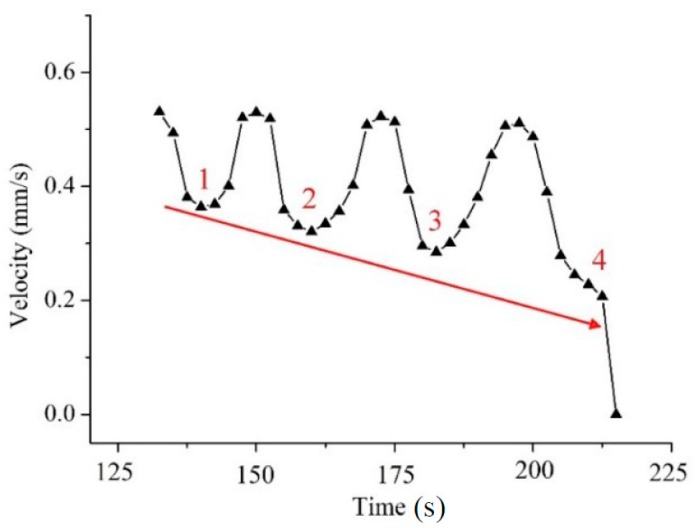
Drawing speed variation of the dieless drawing section, 1–4 represent the drawing speed of the wave peak.

Because the temperature distribution in the dieless drawing deformation zone is stable, the velocity is the key factor affecting the deformation stability. Under the condition that the feeding speed remains constant, the drawing speed of the dieless drawing section plays a decisive role on the deformation stability. When semi-dieless drawing (corresponding to Figure 5) occurs, the drawing speed of the dieless drawing section *v_c_* (which is also the entering speed of the die drawing section) keeps changing during the deformation process, as shown in Figure 8. It can be seen from the figure that the drawing speed of the dieless drawing section first fluctuates and then decreases sharply to 0. When the first fluctuating section of the wire is about to enter the drawing die, the drawing speed of the dieless drawing section is 0.53 mm/s and the speed ratio of the dieless drawing section is 2.12. The die drawing section conforms the volume constant law,

(10) $${v_{o}}^{\prime }D_{die}^{2}=v_{c}D_{c}^{2},$$

when the entering wire diameter *D_c_* increases, the drawing speed *v_o_*′ and wire diameter *D_die_* at the die exit remain constant and the velocity of the entering wire, i.e., the drawing speed of the dieless drawing section *v_c_* decreases.

When the peak of the wire enters the die and deforms, the drawing speed of the dieless drawing section *v_c_* decreases to 0.37 mm/s, and the speed ratio of the dieless drawing section is 1.48. When the entering wire diameter *D_c_* decreases, it is also known from the volume constant law that the drawing speed of the dieless drawing section *v_c_* will increase. When the valley of the wire enters the die and deforms, the drawing speed of the dieless drawing section *v_c_* increases to 0.52 mm/s, and the speed ratio of the dieless drawing section is 2.08. Therefore, when the first fluctuating section of the wire undergoes die drawing, the speed ratio of the dieless drawing section also fluctuates, decreasing from 2.12 to 1.48 and then increasing to 2.08 gradually. The fluctuation of the speed ratio of the dieless drawing section results in the emergence of the third fluctuating section. Similarly, when the second fluctuating section undergoes die drawing, the entering wire velocity decreases from 0.52 to 0.33 mm/s, i.e., the speed ratio of the dieless drawing section decreases from 2.08 to 1.32 gradually. The dieless drawing deformation produces the fourth fluctuating section, and the diameter of the peak wire is larger than that in the third fluctuating section. When the third peak wire enters the die and deforms, the drawing speed of the dieless drawing section is 0.30 mm/s and speed ratio is 1.2. When the fourth peak wire enters the die and deforms, the drawing speed of the dieless drawing section is 0.21 mm/s and speed ratio is 0.84. Subsequently, the wire experiences necking shrinkage at the die exit, dieless drawing stops, and drawing speed of the dieless drawing section decreases to 0.

The above analysis shows that the dieless drawn wire with diameter fluctuation entering the die for die drawing deformation will lead to a fluctuating decreasing trend of the velocity of the entering wire. The velocity of the entering wire is also the drawing speed of the dieless drawing, which causes a fluctuating decrease in the speed ratio of the dieless drawing and fluctuating increase in the entering wire diameter. The stability of the semi-dieless drawing becomes worse. With the progress of the drawing process, when the diameter of the entering wire increases to a certain extent, i.e., the deformation amount of the die drawing exceeds the safe drawing limit, the wire breaks at the die exit, and the semi-dieless drawing process fails.

Therefore, when the drawing speed is low and the deformation amount is small, the diameter fluctuation of the entering wire is small, and the semi-dieless drawing process is stable. When the drawing speed is high and deformation amount is large, the diameter of the entering wire fluctuates significantly, and the semi-dieless drawing process easy loses its stability. To make semi-dieless drawing realize a large deformation amount and remain steady, it is necessary to control the wire diameter fluctuation.

### 3.3. Validation

**Figure 9:**
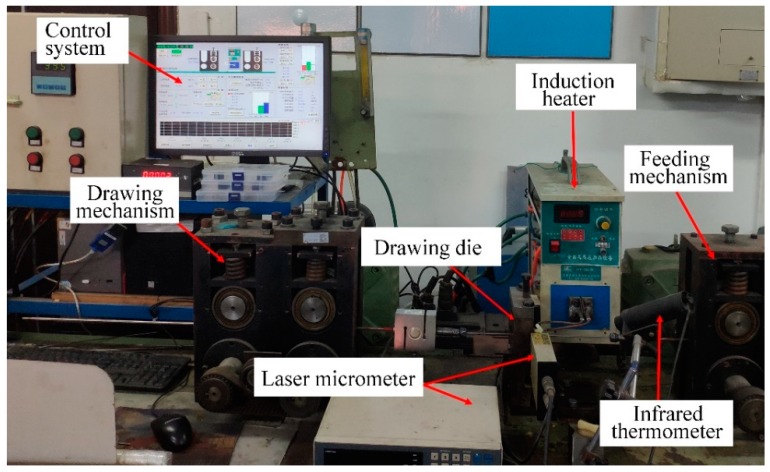
Experimental equipment of the semi-dieless drawing process.

**Figure 10:**
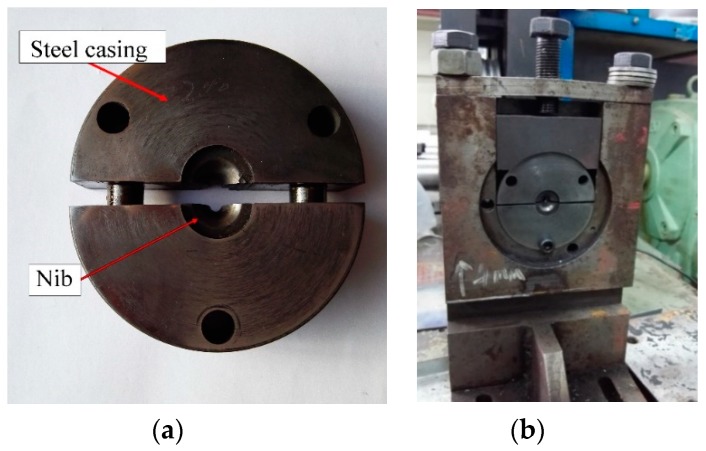
Drawing die: (**a**) separate structure die, (**b**) die closing mechanism.

To verify the correctness of the FE model, semi-dieless drawing experiments are conducted. The experimental equipment of the semi-dieless drawing is shown in Figure 9, which is composed of a high-frequency induction heater, feeding mechanism, drawing mechanism, drawing die, laser micrometer, infrared thermometer, and control system. In the traditional die drawing process, pre-processing treatments such as pointing are required to make the end diameter of the wire smaller than the diameter of the die. Consequently, the metal wire can pass through the drawing die. In this study, a separate structure die is designed to avoid pointing the wire, as shown in Figure 10. First, the drawing die is opened, and the raw wire is passed through the die. Second, the raw wire undergoes dieless drawing for a feeding speed *v_i_* and drawing speed *v_o_*. The diameter of the dieless drawn wire *D_o_* is smaller than diameter of the die *D_die_*. Finally, the die is closed, and the drawing speed is reduced to *v_o_*′ so that diameter of the dieless drawn wire *D_c_* is larger than diameter of the die *D_die_*, and the wire deformation changes from dieless drawing to semi-dieless drawing.

**Figure 11:**
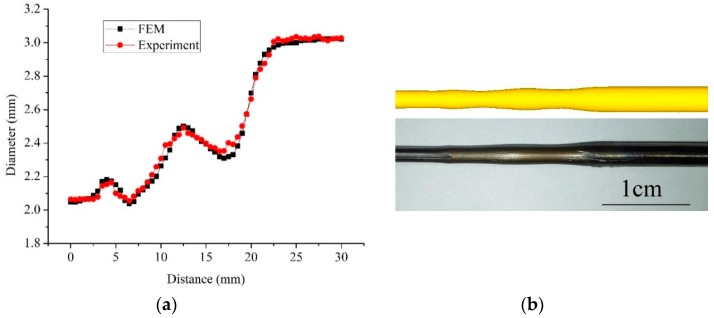
Diameter (**a**) and profile (**b**) of the semi-dieless drawn wire.

The FE simulations and semi-dieless drawing experiments are conducted under the same conditions. The feeding speed is 0.25 mm/s, drawing speed is 0.45 mm/s, heating temperature is 950 °C, the distance between the drawing die and heating coil is 20 mm, and diameter of the die is 2.1 mm. Figure 11 shows the diameter and profile of the wire during the semi-dieless drawing process. It can be seen from the figure that the FE simulation results are consistent with the experiment results, i.e., the FE simulation in this study can be used to simulate the actual semi-dieless drawing process. It can also be seen from the figure that diameter of the dieless drawn wire is fluctuating and disappears after die drawing.

## 4. Conclusions

In this research, the stability of semi-dieless drawing under different process parameters is studied, and the instability mechanism of the semi-dieless drawing process is revealed. The following conclusions are drawn:

(1) The diameter of the entering wire shows a fluctuating increasing trend in the process of semi-dieless drawing. With decreasing diameter of the die or increasing drawing speed, both the diameter of the entering wire and its fluctuation increase, and the semi-dieless drawing process has more probability of becoming unstable.
(2) Temperature and speed are two key factors influencing the stability of semi-dieless drawing. Under the condition of a constant heating temperature, the temperature gradient in the dieless drawing deformation zone is large and the temperature distribution is stable, which is conducive for a stable deformation.
(3) The dieless drawn wire with the fluctuating diameter entering the die for undergoing die drawing deformation will lead to a fluctuating decreasing trend of the entering wire velocity. The velocity of the entering wire is also the drawing speed of the dieless drawing process, so that they have the same variation trend. The diameter of the entering wire shows a fluctuating increasing trend owing to the variation in the drawing speed, which results in instability in the semi-dieless drawing process.
(4) Comparison of the numerically predicted and experimentally measured diameter of the drawn wire verifies the validity of the FE model.

## Figures and Tables

**Table 1:** Diameter of the die corresponding to different deformation amounts in die drawing (mm).

Drawing Speed (mm/s)	Die Drawing Deformation Amount			
	5%	10%	15%	20%
0.38	2.4	2.3	2.25	2.2
0.45	2.2	2.1	2.05	2.0
0.53	2.0	1.95	1.9	1.8
